# Cost-effectiveness of minimal interventional procedures for chronic mechanical low back pain: design of four randomised controlled trials with an economic evaluation

**DOI:** 10.1186/1471-2474-13-260

**Published:** 2012-12-28

**Authors:** Esther T Maas, Johan NS Juch, J George Groeneweg, Raymond WJG Ostelo, Bart W Koes, Arianne P Verhagen, Merel van Raamt, Frank Wille, Frank JPM Huygen, Maurits W van Tulder

**Affiliations:** 1 Department of Health Sciences and the EMGO Institute for Health and Care Research, Faculty of Earth and Life Sciences, VU University Amsterdam, Amsterdam, The Netherlands; 2 Department of Anaesthesiology, Erasmus Medical Centre, Rotterdam, The Netherlands; 3 Department of Epidemiology and Biostatistics, EMGO Institute for Health and Care Research, VU, University Medical Center Amsterdam, Amsterdam, The Netherlands; 4 Department of General Practice, Erasmus Medical Centre, Rotterdam, The Netherlands; 5 Sport Medical Center Papendal, Arnhem, The Netherlands; 6 Department of Anaesthesiology, Diakonessenhuis, Utrecht/Zeist, The Netherlands

**Keywords:** Chronic mechanical low back pain, Minimal interventional procedures, Multidisciplinary pain programme, Economic evaluation

## Abstract

**Background:**

Minimal interventional procedures are frequently applied in patients with mechanical low back pain which is defined as pain presumably resulting from single sources: facet, disc, sacroiliac joint or a combination of these. Usually, these minimal interventional procedures are an integral part of a multidisciplinary pain programme. A recent systematic review issued by the Dutch Health Insurance Council showed that the effectiveness of these procedures for the total group of patients with chronic low back pain is yet unclear and cost-effectiveness unknown. The aim of the study is to evaluate whether a multidisciplinary pain programme with minimal interventional procedures is cost-effective compared to the multidisciplinary pain programme alone for patients with chronic mechanical low back pain who did not respond to conservative primary care and were referred to a pain clinic.

**Methods:**

All patients with chronic low back pain who are referred to one of the 13 participating pain clinics will be asked to participate in an observational study. Patients with a suspected diagnosis of facet, disc or sacroiliac joint problems will receive a diagnostic block to confirm this diagnosis. If confirmed, they will be asked to participate in a Randomized Controlled Trial (RCT). For each single source a separate RCT will be conducted. Patients with a combination of facet, disc or sacroiliac joint problems will be invited for participation in a RCT as well. An economic evaluation from a societal perspective will be performed alongside these four RCTs. Patients will complete questionnaires at baseline, 3 and 6 weeks, 3, 6, 9 and 12 months after start of the treatment. Costs will be collected using self-completed cost questionnaires.

**Discussion:**

No trials are yet available which have evaluated the cost-effectiveness of minimal interventional procedures in patients with chronic mechanical low back pain, which emphasizes the importance of this study.

**Trial registration number:**

National Trial Register: NTR3531

## Background

Non-specific low back pain is a widespread problem with major social and economical consequences [1,2]. Non-specific low back pain contains 85 – 90% of the low back pain diagnoses and is defined as low back pain not attributable to a recognisable, known specific pathology (e.g. infection, tumour, osteoporosis or fracture) [3-5]. The majority of the patients with low back pain are successfully treated in primary care, approximately 10 – 15% will develop chronic (more than three months) symptoms. In The Netherlands, costs of low back pain were estimated at €3.5 billion in 2007 [6]. Patients developing chronic symptoms are responsible for the majority of these healthcare and socio-economic costs [6]. Additionally, based on demographic developments, it is expected that the number of people with back problems will increase in the coming years [7,8]. Because of the enormous costs of this growing problem, effective interventions aimed at prevention and treatment of chronic low back pain are necessary.

In lower back pain, 80 – 90% is of mechanical origin [9,10]. Mechanical back pain implies that the source of the pain is in the spine or its supporting structures [9]. There is consensus among anaesthesiologists, that minimal interventional procedures are effective for patients with pain presumably resulting from single sources: facet, disc, sacroiliac (SI) joint or a combination of these (defined as mechanical low back pain) [11]. This is at odds with recently performed systematic reviews and multidisciplinary international clinical guidelines. These concluded that there is no strong evidence that supports the effectiveness of minimal interventional procedures in patients with chronic low back pain [12-17]. Most likely due to a lack of Randomised Controlled Trials (RCTs) with a low risk of bias and adequate sample size [12,18]. A recent systematic review issued by the Dutch Health Insurance Council (CVZ) showed that the effectiveness of minimal interventional procedures for the total group of patients with chronic low back pain is unclear and the cost-effectiveness unknown [19]. Based on this lack of evidence, CVZ advised the Ministry of Health in The Netherlands not to reimburse minimal interventional procedures for patient with chronic low back pain within the Dutch public health insurance system. The anaesthesiologists standpoint is that they merely treat a subgroup of patients: patients with mechanical low back pain, for which they argue that minimal interventional procedure would be effective [11].

### Aim

The aim of this project will be to provide a valid, reliable and precise answer to the question whether minimal interventional procedures supplementary to a multidisciplinary pain programme for patients with chronic mechanical low back pain who are referred to a pain clinic are more effective and cost-effective compared with the multidisciplinary pain programme alone.

## Methods

### Study design

**Figure 1:**
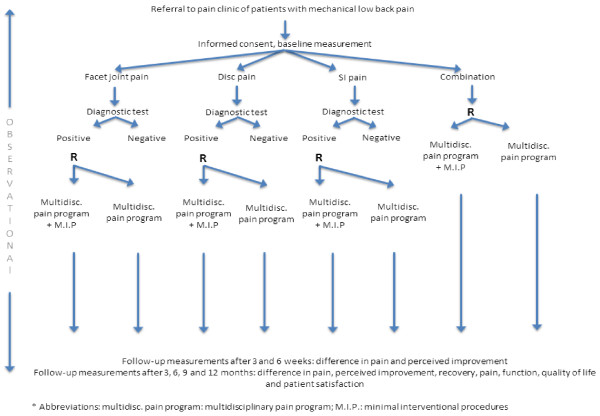
Trial design and patient flow.

An observational study and four RCTs with a full economic evaluation will be performed. The observational data will inform about the proportion of patients with a positive or negative diagnostic test for facet pain, disc pain, SI-joint pain and a combination of these, and the clinical outcomes of patients with a negative diagnostic test. Patients diagnosed with facet, disc, SI-joint or combination pain, by means of a diagnostic block will be asked to take part of one of the four RCTs. The observational study will monitor patients who do not want to, or are not eligible to participate in the RCTs. Figure 1 provides an overview of the study design and patient flow.

### Ethical approval

In June 2012, the study was approved by the Medical Ethics Committee of the Erasmus Medical Centre in Rotterdam (registration number MEC-2012-079) and the study protocol was registered at the Dutch Trial Register (number NTR3531). Written informed consent will be obtained before entering the study.

### Study population

In this study all patients aged between 18 and 70 years, referred to a pain clinic with suspected chronic mechanical low back pain and without improvement of symptoms after conservative treatment will be invited to participate in the observational study. Further inclusion criteria for the RCTs are: a) a 50% or more reduction in perceived pain at 30 minutes after a diagnostic block; or b) the provocative discography must be positive. According to the International Association for the Study of Pain (IASP) and the International Spinal Injection Society (ISIS), a provocative discography is positive when the familiar pain at the target level is provoked with a pain score of 7 out of 10, and two painless adjacent control discs are present [20].

Exclusion criteria are pregnancy; inability to complete the questionnaires; anticoagulant drug therapy and/or coagulopathy; Body Mass Index higher than 35; involvement in a work related conflict; severe psychiatric or psychological problems. Patients will be asked to complete five questionnaires, three of which serve to indicate whether patients with psychiatric or psychological complaints need to be excluded. Distress, depression, anxiety, somatisation, pain acceptance and pain coping strategies will be assessed with the Four-Dimensional Symptoms Questionnaire (4DSQ) [21,22], the Chronic Pain Acceptance Questionnaire (CPAQ) [23,24], Hospital Anxiety Depression Scale (HADS) [25,26], Pain Coping Inventory (PCI) [27], and the Pain Cognition List (PCL) [28].

Only the 4DSQ (anxiety > 12; depression > 5; distress > 20; somatisation > 20), CPAQ (z-score > 2 in pain willingness) and PCL (z-score > 2 in catastrophizing and limitations) will be used for exclusion from the RCTs. Patients will be excluded if they score above the predefined thresh-hold in any of the questionnaires.

Patients are recruited in the pain-treatment clinics of the participating hospitals. General practitioners and medical specialists who referred patients to the pain clinics will be informed about participation of their patients in the study. The anaesthesiologists identify and inform potential participants from among their clinic’s patients. If a patient meets the eligibility criteria and agree to participate in the trial, the patient will be asked to sign an informed consent form.

### Setting

Anaesthesiologists at the participating clinics will conduct the diagnostic tests and the minimal interventional procedures. Every participating pain clinic has a referral agreement with three or four physiotherapy practices in their region. These will provide a standardized exercise programme. The psychological interventions, if necessary, will take place in a usual care setting.

### Multidisciplinary pain programme

Each patient will receive the exercise programme and psychological help (if necessary). The patient in the intervention group will receive the Minimal Interventional Procedures as well. Together, this will form the multidisciplinary pain programme. The selection of eligible patients in the diagnostic phase by performing a test block, as well as the exercise programme (which we standardized for the study) and the minimal interventional procedures are usual care at Dutch pain clinics, as described in the current guideline anaesthesiological pain control [11,29].

#### Exercise programme

All participants of the trials will receive a standardized exercise programme based on the guideline low back pain of the Royal Dutch Society for Physical Therapy [30]. The programme consists of graded exercise therapy with focus on quality of movement (local stabilizers) and behavioural aspects, generally based on the programme of Lindström et al. [31,32]. As the application of these guidelines can vary between physiotherapists, a standardized version of the original programme is developed for this study in cooperation with Sports Medical Center Papendal. All physiotherapists participating in the study have ample experience with exercise therapy with focus on behavioural aspects. Furthermore, all physiotherapists will follow a three to four hour introduction session prior to the study. The main aim of the exercise programme is restoring physical function. An individually graded exercise programme will be offered to the patient explaining that it is safe to move while increasing the level of activity. During the exercise programme the patient has an active role and the physical therapist acts as a coach and supervisor, using a hands-off approach. The entire programme will be spread over three months with a treatment range between eight to twelve hours (one to two sessions per week).

#### Psychological support

If necessary, the anaesthesiologist or physiotherapist can refer the patient to the psychologist. The patients will receive usual care.

#### Minimal interventional procedures

Supplementary to the exercise programme, and possibly a psychological support, the intervention group will receive minimal interventional procedures. This will take place according to a pre-specified approach, based on the current guideline anaesthesiological pain control [11]:

1. Patients with facet joint pain will receive radiofrequency lesion of the first ramus dorsalis at Lumbar vertebrae (L)3, L4, L5 and S1.

2. Patients with intervertebral disc pain will receive Intradiscal Electrothermal Therap (IDET) or Biacuplasty of the involved disc.

3. Patients with SI- joint pain will receive the Cooled radiofrequency, radiofrequency lesion pallisade technique or Simplicity III probe technique of the ramus dorsalis at L4, L5, S1, S2 and S3.

4. Patients with a combination of the single entities will be divided, after the clinical diagnosis, to a group who receives minimal interventional treatments (i.e. a combination of the interventions mentioned under 1, 2 and 3).

In both treatment groups, the patients are asked to refrain from any co-interventions during the intervention period. However, co-interventions after the initial intervention period will be monitored and evaluated. If patients in the non-interventional study group have not improved or recovered after three months, they will not receive interventional procedures but will return to the General Practitioner or medical specialist that had referred them to the pain clinic.

### Baseline measurement & outcomes

The core set of primary outcomes recommended for low back pain research will be used [33]. All web-based questionnaires will be assessed at baseline, three, six, nine and twelve months after the start of the treatment. Quality Adjusted Life Years (QALY), pain intensity and global perceived recovery will be assessed at three and six weeks as well.

#### Baseline measurement

The baseline questionnaire includes socio-demographic characteristics (age, gender, marital status etc.), the complaint history, all primary and secondary outcomes and patient expectation. Patient expectation will be assessed with the 10-point scale Credibility/Expectancy Questionnaire (CEQ) [34,35].

#### Primary outcomes

There are three primary outcomes: pain intensity, global perceived recovery and functional status.

Pain intensity over the previous week will be measured on an 11-point Numerical Rating Scale (NRS) (0 = no pain to 10 = worst imaginable pain) [36]. Among chronic low back pain patients, the NRS is the most appropriate, valid and reliable measurement [37,38].

Global perceived recovery will be measured by self-assessment on a 7-point NRS ranging from ‘completely recovered’ to ‘worse than ever’, as for example described by Kamper et al. [39]. This instrument is often used in low back pain research [35,40].

Functional status will be measured according to the Dutch translation of the Oswestry Disability Index (ODI-NL) [41]. This index consists of ten questions addressing common daily activities. Each question has six answer options, scored 0-5, from which 0 is related to ‘no restriction in daily activities’ to 5 ‘the most restrictions in daily activities’. The ODI has shown to be valid and reliable in chronic pain experience studies and is widely used in low back pain research [42,43].

#### Secondary outcomes

Health-related quality of life will be measured with the EuroQol (EQ-5D) [44]. This questionnaire assesses five dimensions (mobility, self-care, usual activities, pain/discomfort and anxiety/depression) on a 3-point scale (no problems, moderate problems and severe problems). This questionnaire is widely used in cost-utility analyses, and for this purpose applied in the economic evaluation as well [45,46].

Patient satisfaction will be assessed using a written 7-point NRS ranging from ‘not satisfied at all’ to ‘completely satisfied’. No gold standard is available for the measurement of patient satisfaction, but in spinal disorders a seven-point global question is recommended [33].

General health will be evaluated with the Rand-36 [47]. This questionnaire consists of 36 questions, classified in eight subscales: physical functioning, social functioning, role limitations (physical problem), role limitations (emotional problem), mental health, pain, general health perception and health change. Scores (ranging from 0-100) are transformed so that a higher score indicates a better health status. The Dutch translation of the Rand-36 has been validated as well [48].

Chronic pain experiences is measured by the Multidimensional Pain Inventory (MPI) [49]. The MPI contains 61 items divided into three parts. The first measures pain-relevant psychosocial aspects, and contains five scales. In the second part, the responses of the patient's partner to the pain (as perceived by the patient himself) are mapped out. Finally, an inventory is made of the frequency of common daily activities labelled as household chores, outdoor work, activities away from home and social activities, which together form the general activity level. The MPI (and its Dutch Language Version) is a valuable instrument for producing reliable and valid information for therapy-outcome studies with chronic pain patients [50].

#### Economic evaluation

The use of care in the pain clinics will be registered by local research nurses. They will register the type, amount and results of the diagnostic tests and treatments (interventional procedures) the patients receive. Costs of the multidisciplinary pain programme, other health care utilization, patient and family costs, and production losses will be included and relevant data collected using self-completed cost questionnaires [51]. Work absenteeism will be measured with the Productivity and Disease Questionnaire (PRODISC), which includes all relevant aspects of the relationship between health and productivity. The PRODISC was developed and validated in samples of patients and employed people in the Netherlands [52]. Costs will be valued using the guidelines published in the updated handbook for economic evaluations in the Netherlands [53]. Absenteeism from paid work will be valued using the friction cost approach [54].

An overview of the data-collection is presented in Table 1.

**Table 1:** Overview of the data collection

**Outcome measures**		**Follow-up**					
	**Baseline**	**3 weeks**	**6 weeks**	**3 months**	**6 months**	**9 months**	**12 months**
**Baseline measurements**							
Demographic data	x						
Complaint history	x						
Patient expectation (CEQ)	x						
**Primary outcomes**							
Pain Intensity (NRS)	x	x	x	x	x	x	x
Global perceived recovery (NRS)		x	x	x	x	x	x
Functional Status (ODI)	x			x	x	x	x
**Secondary outcomes**							
QALY (EQ-5D)	x	x	x	x	x	x	x
Patiënt satisfaction (NRS)				x	x	x	x
General health (Rand-36)	x			x	x	x	x
Chronic pain experience (MPI)	x			x	x	x	x
**Economic evaluation**							
Costs (diaries)				x	x	x	x
Work absenteeism (PRODISC)				x	x	x	x

Abbreviations: CEQ = Credibility/Expectancy Questionnaire; NRS = Numeric Rating Scale; ODI = Oswestry Disability Index; EQ-5D = EuroQol; MPI = Multidimensional Pain Inventory; PRODISC = Productivity and Disease Questionnaire.

### Sample size

Using a power of .9, alpha .05 and a correlation of .5 for repeated measurements, a total of 85 patients per group are needed to detect a clinically relevant mean difference of two points on the NRS for pain intensity (SD 4) [55]. This difference of two points as Minimal Important Change in the NRS is based on a recent review [55]. Anticipating potential study withdrawal (20%) 102 patients per group or 204 patients per randomized comparison are needed. In total, 816 patients need to be included in the four trials.

Through an ‘educated guess’ a SD of four was chosen, because no SD can be found in the literature. Though arbitrary, it is believed conservative, and will ensure that enough patients will be included to find clinically relevant effects, if present.

### Treatment allocation

Patients who respond positively to the diagnostic test and give informed consent will be randomised to either a group that receives a multidisciplinary pain programme with the minimal interventional procedures, or a group receiving a multidisciplinary pain programme alone. The randomisation will be performed by a local research nurse in each of the participating pain clinics, using a computerised random number generator. The randomisation list is developed centrally. Therefore, the research nurse does not have any influence on the procedure and the treatment allocation is concealed. Randomisation will be stratified for the participating pain clinics. No treatment will be given prior to randomisation.

### Blinding

In this pragmatic trial patients and care providers cannot be blinded. Because all outcome measures are self-reported by the patient, the outcome measurement is not blinded either. Data analysis will be conducted blinded for treatment allocation and blinding will only be finished after the final analyses have been concluded. To ensure data will be analysed anonymously, all patients will be assigned a unique number. To evaluate whether lack of blinding is associated with bias, expectations and preferences of patients will be measured before randomisation and after treatment allocation. Patient satisfaction will be measured after treatment and during follow-up.

### Statistical analysis

Baseline data will be presented comparing the two treatment groups. Intention-to-treat analysis will be conducted for each follow-up moment. Missing data will be imputed with multiple imputation techniques [56,57]. The 95%-confidence intervals will be calculated for the difference of percentages (Chi-square distribution) and means (T-distribution) for dichotomous and continuous outcome variables, respectively. In case of unequal distributions of prognostic factors, multivariate analysis techniques will be used to correct for these between-group differences in prognosis. Multilevel analyses will be performed, with patient, pain clinic, and time of measurement as levels. The threshold of statistical significance is set at *p* < 0.05. Characteristics of patients with missing follow-up data will be compared to those with complete data to identify possible selective drop-out.

### Economic evaluation from a societal perspective

The economic evaluation will be performed according to the intention-to-treat principle and from a societal perspective. Costs of the minimal interventional procedures, physiotherapy, other health care utilization, patient and family costs, and costs of production losses will be measured and valued. Data are collected through self-completed cost questionnaires.

Missing costs and effect data will be imputed using multiple imputation according to the Multivariate Imputation by Chained Equations procedure corrected and accelerated (Bca) bootstrapping with 5000 replications will be used to estimate confidence intervals around differences in total costs between treatment groups [58,59].

A cost-effectiveness and a cost-utility analysis will be performed. Incremental cost-effectiveness ratios will be calculated by dividing the difference in mean costs by the difference in the mean effects of the two treatment groups. Using a primary clinical effect measures of the trial, for instance pain intensity. Cost-utility will be based on the EQ-5D and expressed in costs per QALY. Uncertainty surrounding incremental cost-effectiveness and cost-utility ratios will be estimated using bootstrapping techniques and graphically presented in cost-effectiveness and cost utility planes. Cost-effectiveness acceptability curves and net monetary benefit will also be estimated. Sensitivity analyses on the most important cost drivers will be performed in order to assess the robustness of the results.

## Discussion

The importance of this study is emphasized by the fact that a recent report did not show consensus regarding the effectiveness of minimal interventional procedures for low back pain [60]. This is primarily due to the lack of RCTs with a low risk of bias and an adequate sample size. Conversely, minimal interventional procedures are widely used by anaesthesiologists. Recently published clinical guidelines of the Dutch Society of Anaesthesiologists recommend minimal interventional procedures for a subgroup of patients with low back pain: patients with mechanical low back pain [11]. The decision whether reimbursement of minimal interventional procedures in the Dutch public health insurance system should be continued needs to be made based upon scientific evidence. At this moment it is unclear whether reimbursement should be stopped for the whole group or continued for the subgroup of patients with mechanical low back pain. These four RCTs, including an economic evaluation are designed to provide in this lacking information.

At present, one of the applicants of this study protocol supervises three randomised, double-blind trials that evaluate the efficacy of 1) radiofrequency denervation of the first ramus dorsalis at L3, L4, L5 and S1 in patients with facet joint pain, of 2) radiofrequency denervation of the ramus communicans in patients with intervertebral disc pain, and of 3) radiofrequency denervation of the ramus dorsalis at L5, S1, S2 in patients with SI joint pain. These three trials are placebo controlled and do not include economic evaluations. The current trials will be the first on this topic including an economic evaluation.

We did not choose to perform placebo-controlled experiments. However, because of the nature of the study, it was not desired to perform a placebo-controlled study either [61]. Furthermore, both the ethics and feasibility of performing placebo injections are currently under debate [62,63]. In our study it will not be possible to determine to what extent any observed benefits of the minimal interventional procedures are attributable solely to its placebo effect, which can be considered a limitation of this trial. The pragmatic nature of our RCTs mirrors daily practice which will improve the generalizability [64].

Based on clinical experience we hypothesize that for patients with chronic mechanical low back pain, a multidisciplinary pain programme with minimal interventional procedures will be more effective than a programme without these procedures. The main expected effect is short-term pain reduction, but it is likely that this will be associated with improved functioning and quality of life. The costs of minimal interventional procedures are approximately (depending on the treating hospital and the health insurance company) between €2500,- and €3500,- per treatment [65]. We expect that the average costs of patients who receive minimal interventional procedures are higher than those of patients in the control group. However, if clinical outcomes are better minimal interventional procedures may be cost-effective if the incremental costs are worthwhile. Also, improved clinical outcomes and more rapid recovery might result in less additional health care consumption and lower costs of work absenteeism and consequently in lower additional costs.

This study will provide more knowledge on the treatment of chronic mechanical low back pain. Furthermore, results of this project will be included in future updates of the clinical guidelines of low back pain. The Dutch Association of Anaesthesiology supports this project and will use the results in a future update of their clinical guidelines. Finally, the results of this study will inform governmental policy regarding reimbursement of minimal interventional procedures for chronic mechanical low back pain. The results of the trials are expected to be available at the end of 2015.

## Abbreviations

RCT: Randomized controlled trial; SI-joint: Sacroiliac joint; CVZ: Health insurance council; IASP: International association for the study of pain; ISIS: International spinal injection society; 4DSQ: Four-dimensional symptoms questionnaire; CPAQ: Chronic pain acceptance questionnaire; HADS: Hospital anxiety depression scale; PCI: Pain coping inventory; PCL: Pain cognition list; L: Lumbar vertebrae; IDET: Intradiscal electrothermal therapy; QALY: Quality adjusted life years; CEQ: Credibility/expectancy questionnaire; NRS: Numerical rating scale; ODI: Oswestry disability index; EQ-5D: EuroQol; MPI: Multidimensional pain inventory; PRODISC: Productivity and disease questionnaire; SD: Standard deviation.

## Competing interests

The authors affirm that this study has not received any funding/assistance from a commercial organization and we do not keep any commercial relationships which may lead to a conflict of interests. The Netherlands Society of Anesthesiologists (NVA) finances the data management for the studies. The NVA will use the data management programme for quality registration when the studies are finished. A member of the Netherlands Society of Anesthesiologists (Frank Wille) is represented in the project group.

## Authors’ contributions

All authors have been involved in the development of the study design and research protocols. All authors read and corrected draft versions of the manuscript and approved the final manuscript.

## Pre-publication history

The pre-publication history for this paper can be accessed here:

http://www.biomedcentral.com/1471-2474/13/260/prepub
